# Supplementary material for: Too old to telework? Age but not gender shapes hiring biases across telework and office settings
Source: PLoS One. 2026 Jan 20;21(1):e0340366. doi: 10.1371/journal.pone.0340366 (PMC12818692; doi:10.1371/journal.pone.0340366)
Supplement: S2 File — (DOCX) [file pone.0340366.s002.docx]

**Research Materials**

**Study 1**

Below you will read a description of a situation at work. Please read the story carefully and try to place yourself in that situation. It is important that you try to immerse yourself fully into that situation. Try to imagine what you would feel, think and do if you were in that situation.

**Scenario**

Imagine that you work as HR manager in a big company in Amsterdam. In your company a job vacancy is open and you are responsible for selecting the most suitable candidate. Please take a few seconds to put yourself in the shoes of the job recruiter and to immerse yourself into this role.

**Job description**

Your company is looking for a "Project Manager".

A Project Manager's duties involve coordinating a team of 10 to 20 people. You are looking for a *decisive person with analytical ability* who monitors the project progress. Moreover, the selected candidate should be able to develop *good relationships* with the team members and the customers.

**Work Setting Manipulation**

***Office Setting Manipulation***

It is important to mention that the successful candidate will work 35-40 hours per week *from the company office*. *Physical presence of the candidate in the office is mandatory*. All kinds of formal or informal meetings, business discussions, and social interactions with team members and customers will be *face-to-face*. This means that the successful candidate will only meet other people *face-to-face* while no online interactions will take place. In other words, the successful candidate will be working with people that he or she will be able to meet in real life. Teleworking is not an option in the company.

***Working From Home Manipulation***

It is important to mention that the successful candidate will work 35-40 hours per week *remotely (e.g., from home). The physical presence of the candidate in the company is not necessary*. All kinds of formal or informal meetings, business discussions, and social interactions with team members and customers will be *completely online*. This means that the successful candidate will *only meet other people virtually while no face-to-face- interactions will take place*. In other words, the successful candidate will be working with people that he or she might never be able to meet in real life. Working from a company ofﬁce is not an option in the company.

**Gender and Age of Candidates Manipulations**

Imagine that based on the applications that you received and the candidates' qualifications, *four candidates have been shortlisted.*
 You are going to be presented with a short description *of the background of one of the four candidates.*
 The candidate that you will read about has been randomly chosen out of the list of the four shortlisted candidates. Other participants might read about a different candidate.
In a nutshell, four candidates have been shortlisted in total, including the one that you will read about. Move to the next page to read a short description of the background of one of the four shortlisted candidates. Then we will ask you to give us your assessment of that candidate.

***Older Female Candidate***

Ms Johanna de Vries was born in 1968 and she, therefore, is *55 years old.* This is the candidate, Ms Johanna de Vries.

--------------------Photo of a 55-year old looking female follows--------------------------

Ms de Vries has considerable experience in Project Management. Based on her CV, Ms de Vries fulfils the job requirements. However, the company would need to invest time and money in order to provide Ms de Vries the necessary training to enable her to perform as expected. Although training newcomers is always necessary, it seems that some extra time and money investment is needed in case that Ms de Vries gets hired.

***Older Male Candidate***

Mr Jan de Vries was born in 1968 and he, therefore, is *55 years old.* This is the candidate, Mr Jan de Vries.

--------------------Photo of a 55-year old looking male follows--------------------------

Mr de Vries has considerable experience in Project Management. Based on his CV, Mr de Vries fulfils the job requirements. However, the company would need to invest time and money in order to provide Mr de Vries the necessary training to enable him to perform as expected. Although training newcomers is always necessary, it seems that some extra time and money investment is needed in case that Mr de Vries gets hired.

***Younger Female Candidate***

Young-female Ms Johanna de Vries was born in 1993 and she, therefore, is *30 years old.* This is the candidate, Ms Johanna de Vries.

--------------------Photo of a 30-year old looking female follows--------------------------

Ms de Vries has considerable experience in Project Management. Based on her CV, Ms de Vries fulfils the job requirements. However, the company would need to invest time and money in order to provide Ms de Vries the necessary training to enable her to perform as expected.

Although training newcomers is always necessary, it seems that some extra time and money investment is needed in case that Ms de Vries gets hired.

***Younger Male Candidate***

Mr Jan de Vries was born in 1993 and he, therefore, is *30 years old*. This is the candidate, Mr Jan de Vries.

--------------------Photo of a 30-year old looking male follows--------------------------

Mr de Vries has considerable experience in Project Management. Based on his CV, Mr de Vries fulfils the job requirements. However, the company would need to invest time and money in order to provide Mr de Vries the necessary training to enable him to perform as expected. Although training newcomers is always necessary, it seems that some extra time and money investment is needed in case that Mr de Vries gets hired.

**Measures**

***Manipulation Check Work Setting***

Now that you have a clear picture in your mind regarding the job description we would like to ask you two short questions about it. Based on what you just read, the successful candidate will work…

- Completely remotely from home (1 = *Not at all true*, 7 = *Completely true*).
- Always from the company office (1 = *Not at all true*, 7 = *Completely true*).

***Manipulation Check Age***

Please try to recall the description of the candidate that you read and answer the following simple questions. Your answers will help us understand if you read the description carefully:

The candidate that you just read about is… (participants could select one of the two options)

- 30 years old
- 55 years old

***Perceived Warmth and Competence of the Applicant (example female candidate)***

Bring to mind the candidate that was described in the scenario that you read. Take a few seconds to think about her as a person and bring to mind her qualities and traits. To what extent do you think Ms de Vries can demonstrate each of the following characteristics while at work?

We acknowledge that we have not given you much information about the candidate. Yet, we would appreciate your assessment of her based on 1) what you read about her and 2) the picture of her that you saw.

Based on the short description I read about the candidate and the picture of the candidate that I saw, I would say that Ms De Vries strikes me as a person who is... (participants were asked to rate the following items from 1 = *Not at all* to 7 = *To a great extent;* items were presented in randomized order)

- Efficient
- Active
- Capable
- Energetic
- Competent
- Skillful
- Intelligent
- Warm
- Friendly
- Helpful
- Supportive
- Kind
- Sociable
- Likable

***Hiring Recommendation***

Now we ask you to indicate whether you would recommend the candidate for the position of Project Manager at your company.

Based on what you read about Ms de Vries [Mr de vries]...

- Would you recommend that Ms de Vries [Mr. de Vries] be hired? (1 = *Absolutely not*, 7 = *Absolutely yes*)
- What is the likelihood that you would recommend Ms de Vries [Mr. de Vries] for hiring by your company? (1 = *Very unlikely*, 7 = *Very likely*)

**Study 2**

**Scenario**

Imagine that you work as HR manager in a big company in Amsterdam. In your company a job vacancy is open and you are responsible for selecting the most suitable candidate. Please take a few seconds to put yourself in the shoes of the job recruiter and to immerse yourself into this role.

**Job Description**

You are currently looking for a "Project Coordinator" to join your team. He/she will be responsible for coordinating project activities, managing timelines, and facilitating communication and interaction among team members. His/her role will require a combination of organizational competence to ensure projects stay on track and effective interpersonal skills to foster collaboration and teamwork.

**Work Setting Manipulation:** Same as Study 1

**Gender and Age of Candidates Manipulations**

***Older Female Candidate***

Ms. Johanna de Vries was born in 1964 and she, therefore, is 60 years old. This is the candidate, Ms. Johanna de Vries.

--------------------Photo of a 60-year old looking female follows--------------------------

Ms. de Vries has considerable experience in Project Coordination. Based on her CV, Ms. de Vries fulfils the job requirements. However, the company would need to invest time and money in order to provide Ms. de Vries the necessary training to enable her to perform as expected. Although training newcomers is always necessary, it seems that some extra time and money investment is needed in case that Ms. de Vries gets hired.

***Older Male Candidate***

Mr. Jan de Vries was born in 1964 and he, therefore, is*60 years old.* This is the candidate, Mr. Jan de Vries. 
--------------------Photo of a 60-year old looking male follows--------------------------

Mr. de Vries has considerable experience in Project Coordination. Based on his CV, Mr. de Vries fulfils the job requirements. However, the company would need to invest time and money in order to provide Mr. de Vries the necessary training to enable him to perform as expected. Although training newcomers is always necessary, it seems that some extra time and money investment is needed in case that Mr. de Vries gets hired.

***Younger Female Candidate***

Ms Johanna de Vries was born in 1996 and she, therefore, is 28 years old. This is the candidate, Ms Johanna de Vries.
--------------------Photo of a 28-year old looking female follows--------------------------

Ms de Vries has considerable experience in Project Coordination. Based on her CV, Ms de Vries fulfils the job requirements. However, the company would need to invest time and money in order to provide Ms de Vries the necessary training to enable her to perform as expected. Although training newcomers is always necessary, it seems that some extra time and money investment is needed in case that Ms de Vries gets hired.

***Younger Male Candidate***

Mr. Jan de Vries was born in 1996 and he, therefore, is*28 years old.* This is the candidate, Mr. Jan de Vries. 
--------------------Photo of a 28-year old looking male follows--------------------------

Mr. de Vries has considerable experience in Project Coordination. Based on his CV, Mr. de Vries fulfils the job requirements. However, the company would need to invest time and money in order to provide Mr. de Vries the necessary training to enable him to perform as expected. Although training newcomers is always necessary, it seems that some extra time and money investment is needed in case that Mr. de Vries gets hired.

**Measures:** Same as in Study 1

**Study 3**

**Materials Study 3**

Below you will read a description of a situation at work. Please read the story carefully and try to place yourself in that situation. It is important that you try to immerse yourself fully into that situation. Try to imagine what you would feel, think and do if you were in that situation.

**Scenario
 Imagine that you work as HR manager in a big company in Amsterdam. In your company a job vacancy is open and you are responsible for selecting the most suitable candidate. Please take a few seconds to put yourself in the shoes of the job recruiter and to immerse yourself into this role.**

Job description:
You are currently looking for a "**Project Coordinator**" to join your team.
The successful candidate will be responsible for coordinating project activities, managing timelines, and facilitating communication and interaction among team members. His/her role will require a combination of organizational competence to ensure projects stay on track and effective interpersonal skills to foster collaboration and teamwork.

**Manipulation Work Setting**

***Office Setting Manipulation***

It is important to mention that the successful candidate will work 35-40 hours per week*from the company office. Physical presence of the candidate in the office is mandatory.*All kinds of formal or informal meetings, business discussions, and social interactions with team members and customers will be *face-to-face*. This means that the successful candidate will only meet other people *face-to-face* while no online interactions will take place. In other words, the successful candidate will be working with people that he or she will be able to meet in real life. Teleworking is not an option in the company.

***Working From Home Manipulation***

It is important to mention that the successful candidate will work 35-40 hours per week remotely (e.g., from home). Physical presence of the candidate in the company is not encouraged. All kinds of formal or informal meetings, business discussions, and social interactions with team members and customers will be completely online. This means that the successful candidate will only meet other people virtually while no face-to-face-interactions will take place. In other words, the successful candidate will be working with people that he or she might never be able to meet in real life. Working from a company office is not an option in the company.

**Manipulation checks**

Now that you have a clear picture in your mind regarding the job description we would like to ask you two short questions about it.

Based on what you just read, the successful candidate will work… (1=not at all true, 7 = completely true)

- Completely remotely from home…
- Always from the company office…

**Candidates**

Imagine that based on the applications that you received and the candidates' qualifications, *four candidates have been shortlisted.*

**You are going to be presented with a short description *of the background of one of the four candidates****(For anonymity reasons, in this survey we address candidates as Candidate A, B, C, D).*

**The candidate that you will read about has been randomly chosen out of the list of the shortlisted candidates. Other participants might read about a different candidate.**
Move to the next page to read a short description of the background of one of the four shortlisted candidates. Then we will ask you to give us your assessment of that candidate.

***Manipulation Older Candidate***

**You will read the committee's notes about Candidate A.**

**Candidate A was born in 1965 and this candidate, therefore, is*60 years old.***

Candidate A has experience in Project Coordination and based on his/her CV, the candidate seems to fulfil the job requirements. While some training and adjustment will be necessary, as with any new hire, Candidate A is likely to be ready to start the role soon.

Please note that three additional candidates have been shortlisted, but you will not have access to the hiring committee's notes on them. As a result, you cannot determine whether any of their CVs are stronger or weaker than Candidate A’s.

***Manipulation Mid-aged Candidate***

**You will read the committee's notes about Candidate A.
Candidate A was born in 1980 and this candidate, therefore, is*45 years old.***

Candidate A has experience in Project Coordination and based on his/her CV, the candidate seems to fulfil the job requirements. While some training and adjustment will be necessary, as with any new hire, Candidate A is likely to be ready to start the role soon.

Please note that three additional candidates have been shortlisted, but you will not have access to the hiring committee's notes on them. As a result, you cannot determine whether any of their CVs are stronger or weaker than Candidate A’s.

***Manipulation Younger Candidate***

**You will read the committee's notes about Candidate A.**

**Candidate A was born in 1997 and this candidate, therefore, is *28 years old*.**

Candidate A has experience in Project Coordination and based on his/her CV, the candidate seems to fulfil the job requirements. While some training and adjustment will be necessary, as with any new hire, Candidate A is likely to be ready to start the role soon.

Please note that three additional candidates have been shortlisted, but you will not have access to the hiring committee's notes on them. As a result, you cannot determine whether any of their CVs are stronger or weaker than Candidate A’s.
 
**Manipulation checks**
Please try to recall the description of the candidate that you read and answer the following simple questions. Your answers will help us understand if you read the description carefully:
The candidate that you just read about is…

- 60 years old
- 45 years old
- 28 years old

Bring to mind the candidate that was described in the scenario that you read. Take a few seconds to think about him/her as a person.
To what extent do you think Candidate A can demonstrate each of the following characteristics while at work?
We acknowledge that we have not given you much information about the candidate. Yet, we would appreciate your assessment of Candidate A based on what you read about his/her.

**Warmth**

Based on the short description I read, I would say that while at work, Candidate A is likely to be... (1= Not at all, 7 = a great deal)

- Likable
- Supportive
- Helpful
- Social
- Friendly
- Kind
- Warm

**Competence**

Based on the short description I read, I would say that while at work, Candidate A is likely to be... (1= Not at all, 7 = a great deal)

- Active
- Skillful
- Capable
- Intelligent
- Competent
- Energetic
- Efficient

**Hiring recommendation**

Now we ask you to indicate whether you would recommend Candidate A for the position of Project Coordinator at your company.

Based on what you read about Candidate A...

- Would you recommend that Candidate A be hired? (1 = Absolutely no, 7 = Absolutely yes)
- What is the likelihood that you would recommend Candidate A for hiring by your company/department? (1 = Absolutely not, 7 = Absolutely yes)

**Home-work Interference**

Although we do not expect that you have gotten a complete picture of the profile of Candidate A, we would still like to ask you the following:

**Based on the short description of Candidate A in the scenario that you just read, to what extent would you expect (or guess) that...**

- The job requirements of this position would interfere with Candidate A's responsibilities at home, such as cooking, shopping, child care, yard work, and house repairs
- The demands of the job would interfere with Candidate A's home and family life
- The job requirements of this position would prevent Candidate A from spending the desired amount of time with his/her family
- The job would produce strain that would make it difficult for Candidate A to fulfil his/her family duties
- Home life would interfere with Candidate A's responsibilities at work, such as getting to work on time, accomplishing daily tasks or working overtime
- Candidate A's home life would prevent him/her from spending the desired amount of time on job- or career-related activities
- To show that you are paying attention, please choose "7 = To a great extent" for this question (Attention check)

**Digital Skills**

Based on the description of the candidate that you read, to what extent you agree with the following statements? (1 = strongly disagree, 7 = strongly agree)

- Candidate A is likely to make good use of digital technologies for the communication with his/her colleagues.
- Candidate A likely makes poor use of digital technologies for the collaboration with his/her colleagues. (Recode)
- Candidate A likely makes good use of digital technologies for sharing information with his/her colleagues.

**Demographics**

What is your age? (in years)

What is your gender? (male, female, other, prefer not to say)

Please indicate your nationality:
